# Parvalbumin-expressing interneurons can act solo while somatostatin-expressing interneurons act in chorus in most cases on cortical pyramidal cells

**DOI:** 10.1038/s41598-017-12958-4

**Published:** 2017-10-06

**Authors:** Mir-Shahram Safari, Javad Mirnajafi-Zadeh, Hiroyuki Hioki, Tadaharu Tsumoto

**Affiliations:** 1 Brain Science Institute, RIKEN, Wako, 351-0198 Japan; 2 Neuroscience Research Center, Shahid Beheshti University of Medical Sciences, Tehran, 19615-1178 Iran; 3 Department of Physiology, Faculty of Medical Sciences, Tarbiat Modares University, Tehran, 14117-13116 Iran; 4 Department of Morphological Brain Science, Graduate School of Medicine, Kyoto University, Kyoto, 606-8501 Japan

## Abstract

Neural circuits in the cerebral cortex consist primarily of excitatory pyramidal (Pyr) cells and inhibitory interneurons. Interneurons are divided into several subtypes, in which the two major groups are those expressing parvalbumin (PV) or somatostatin (SOM). These subtypes of interneurons are reported to play distinct roles in tuning and/or gain of visual response of pyramidal cells in the visual cortex. It remains unclear whether there is any quantitative and functional difference between the PV → Pyr and SOM → Pyr connections. We compared unitary inhibitory postsynaptic currents (uIPSCs) evoked by electrophysiological activation of single presynaptic interneurons with population IPSCs evoked by photo-activation of a mass of interneurons *in vivo* and *in vitro* in transgenic mice in which PV or SOM neurons expressed channelrhodopsin-2, and found that at least about 14 PV neurons made strong connections with a postsynaptic Pyr cell while a much larger number of SOM neurons made weak connections. Activation or suppression of single PV neurons modified visual responses of postsynaptic Pyr cells in 6 of 7 pairs whereas that of single SOM neurons showed no significant modification in 8 of 11 pairs, suggesting that PV neurons can act solo whereas most of SOM neurons may act in chorus on Pyr cells.

## Introduction

In the cerebral neocortex GABAergic/inhibitory interneurons control tuning and/or gain of response of pyramidal (Pyr) cells to sensory stimuli^1–6^. GABAergic interneurons are divided into several subtypes, in which the two major groups in the rodent neocortex are those expressing parvalbumin (PV) or somatostatin (SOM)^7–16^. Recent studies in the mouse visual cortex reported notable differences in function between these subtypes of interneurons, such as scaling visual responses through divisive inhibition or sharpening response tuning through subtractive inhibition^17–19^, although there is some inconsistency among these reports^20^. Also it is suggested that PV-expressing and SOM-expressing interneurons control response reliability in a different way^21^. It is not fully clear, however, why their functions are so different and what mechanisms in cortical circuits underlie the different functions.

To understand mechanisms underlying the different functional roles of these interneurons in cortical circuits, the quantitative information on functional connectivity with target Pyr cells is crucial. However, there has been no solid information about how many PV or SOM interneurons make functional connections with a postsynaptic Pyr cell, although some quantitative analysis was made previously^22–24^. By combining the methods of optogenetic, massive cell activation with those of electrophysiological single cell activation, we addressed these questions and found that about 14 PV neurons at least made strong connections with a postsynaptic Pyr cell while a much larger number of SOM neurons made weak connections. The activation/inactivation of single PV neurons modified visual responses of postsynaptic Pyr cells in 6 of the 7 pairs whereas that of single SOM neurons did not induce such a modification in 8 of the 11 pairs, suggesting that the operation mode of the two major subtypes of interneurons is different.

## Results

### Difference in IPSCs between PV → Pyr and SOM → Pyr cell connections *in vivo*

**Figure 1:**
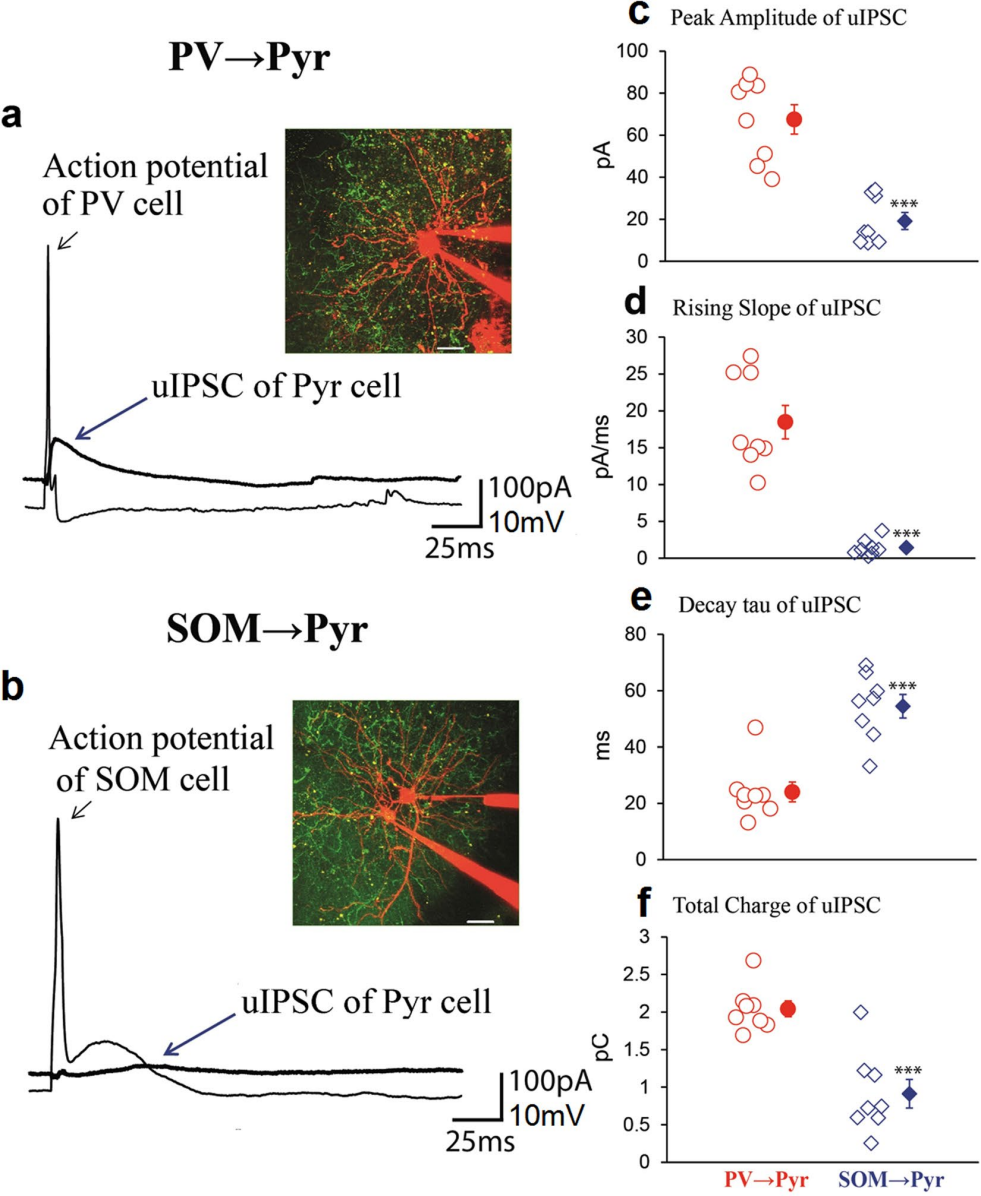
Unitary IPSC (uIPSC) of Pyr cells evoked by action potentials of PV or SOM neurons *in vivo*. (**a**) A PV neuron generated action potential which induced uIPSC in a Pyr cell, as indicated by arrows. Arrangement of the two cells and electrodes is shown in inset. The distance between the soma of the two cells was 26 μm. The cell bodies and dendrites are visualized by Alexa. Green signals mostly represent dendrites of other PV cells which expressed ChR2. Scale at bottom, 20 μm. (**b**) A SOM neuron generated action potential which induced uIPSC in a Pyr cell, as indicated by arrows. Other conventions are the same as in (**a**). (**c**–**f**) Peak amplitude, rising slope, decay tau and total charge of uIPSCs of 8 Pyr cells evoked by action potentials of PV neurons (left) and those of another 8 Pyr cells evoked by action potentials of SOM neurons (right). Filled symbol with vertical bar in the right represents means ± SEM. In a few cases the value of SEM was very small so that it did not appear outside the symbol. Triple asterisks indicate that the difference in the mean between the left and right columns is statistically significant at *p* < 0.001 (unpaired *t*-test).

Initially we analyzed inhibitory postsynaptic currents (IPSCs) of Pyr cells evoked by action potentials of presynaptic PV or SOM neurons *in vivo* in layer 2/3 of the visual cortex of mice in which each subtype of interneurons expressed channelrhodopsin-2 (ChR2). To activate single PV or SOM neurons we injected depolarizing currents into target interneurons through recording electrodes so as to generate action potentials which induced unitary IPSCs (uIPSCs) in postsynaptic Pyr cells. The intensity of injected currents was adjusted to generate single action potentials. We found that there were notable differences in uIPSCs between PV → Pyr and SOM → Pyr cell connections. In a pair of PV and Pyr cells uIPSCs had relatively high amplitudes and fast rising slopes (Fig. 1a). The peak amplitude, the rising slope, the decay tau and the total charge of currents were 84 pA, 27 pA/ms, 25 ms and 1.9 pC, respectively. On the other hand, action potentials of SOM interneurons induced very small uIPSCs in postsynaptic Pyr cells. In the pair shown in Fig. 1b the values were 14 pA, 0.6 pA/ms, 49.4 ms and 0.6 pC, respectively. The differences between PV neuron- and SOM neuron-induced uIPSCs were confirmed by the group analysis. The mean peak amplitude of uIPSCs of 8 PV → Pyr cell pairs was 67.5 ± 7.0 (SEM) pA while that of another 8 SOM → Pyr cell pairs was 19.2 ± 4.0 pA (Fig. 1c). The mean rising slope, decay tau and total charge of uIPSCs of the 8 PV → Pyr cell pairs were 18.5 ± 2.3 pA/ms, 24.1 ± 3.5 ms and 2.0 ± 0.1 pC, respectively while those of the 8 SOM → Pyr cell pairs were 1.4 ± 0.3 pA/ms, 54.5 ± 4.2 ms and 0.9 ± 0.2 pC (Fig. 1d–f).

Then we activated a population of PV or SOM neurons which make functional connections with a target Pyr cell under observation by emission of blue laser to the cortex through the microscope. An application of laser pulse of 1 ms width faithfully generated single action potentials of interneurons which in turn evoked IPSCs in postsynaptic Pyr cells (Supplementary Fig. S1a–f). The effectiveness of blue laser was confirmed by the observation that phase-locked single action potentials were evoked by each emission in all of the 12 PV and 11 SOM neurons tested. There was no notable jitter in the latency from laser pulse to action potential (see Supplementary Fig. S1g,h,j). We changed the duration of blue laser from 0.1 to 10 ms and the intensity of laser from 10–20 mW/mm^2^ to find parameters for generation of single action potentials. We found that the optimal intensity was 18 mW/mm^2^ for 1 ms width both for PV and SOM neurons, and there was no detectable difference in sensitivity to blue laser between PV and SOM neurons. In part of the experiments we increased the intensity to 20 mW/mm^2^ that did not induce multiple action potentials. We also observed that none of the 8 PV- or 7 SOM-negative neurons that were presumed to be interneurons on the basis of their soma shape generated action potentials in response to blue laser. Also we confirmed that none of the 11 GAD-positive but ChR2-negative cells showed action potentials to photoactivation. These results suggested that the photoactivation selectively generated action potentials of PV or SOM interneurons.

**Figure 2:**
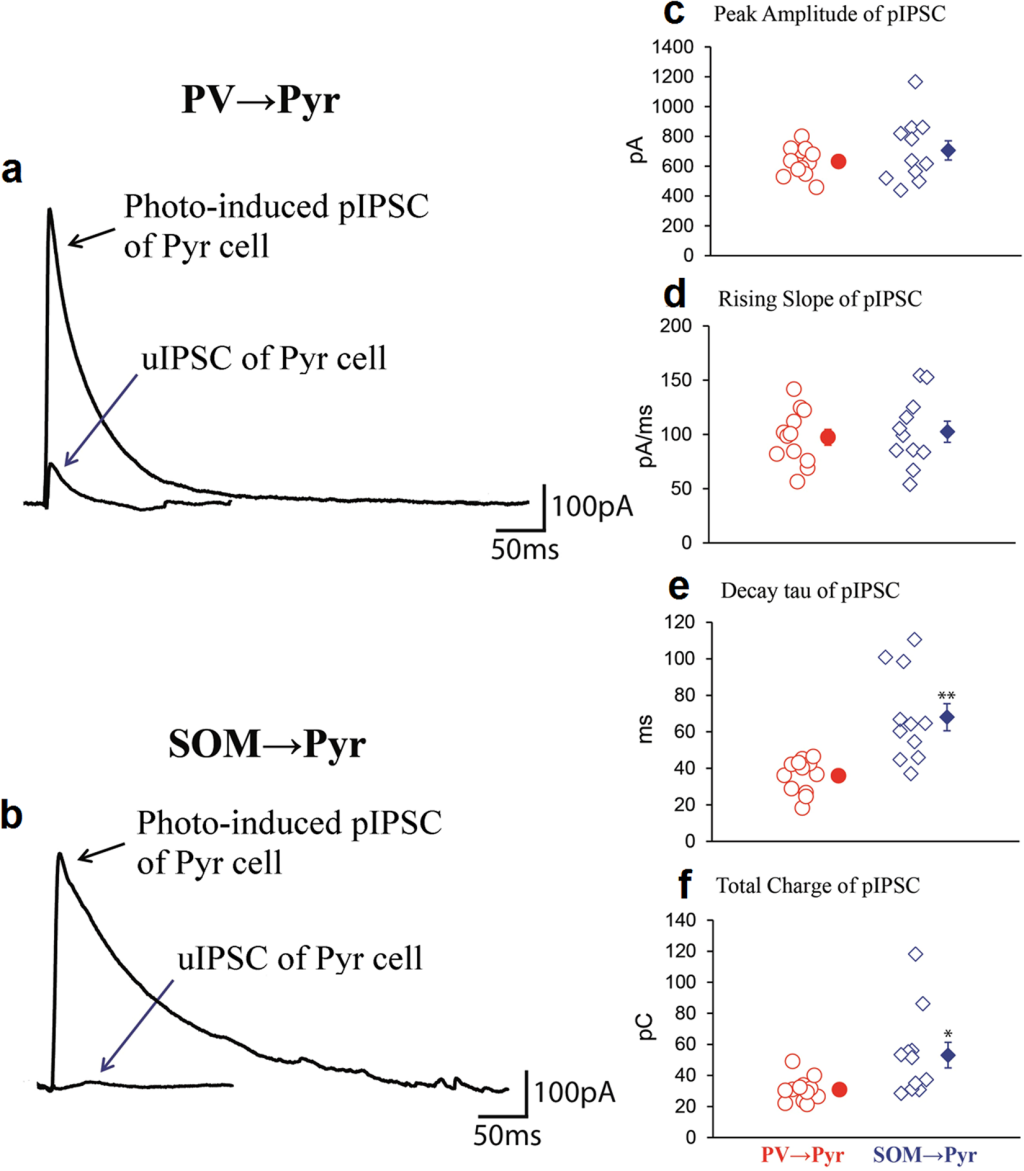
Population IPSC (pIPSC) evoked by photoactivation of a mass of PV or SOM neurons. (**a**,**b**) pIPSCs recorded from the same neurons as in Fig. 1a and b, respectively, so that the traces are superimposed with the uIPSCs shown in Fig. 1 at the slower time scale. (**c**–**f**) Peak amplitude, rising slope, decay tau and total charge of pIPSCs of 12 Pyr cells evoked by the photoactivation of PV neurons (left) and those of another 11 Pyr cells evoked by the photoactivation of SOM neurons (right). Double and single asterisks indicate that the difference in the mean between the left and right columns is statistically significant at *p* < 0.01 and *p* < 0.05, respectively (unpaired *t*-test).

Because almost all interneurons of the same subtype expressed ChR2 (Supplementary Fig. S2), blue laser which illuminated the volume of about 200 × 200 × 400 μm of the cortex was expected to simultaneously activate a population of PV or SOM neurons surrounding the target Pyr cell. In fact, it generated huge IPSCs (population IPSCs, or pIPSCs) in postsynaptic Pyr cells (Fig. 2a,b). These pIPSCs seemed to be saturated because blue light at the slightly weaker intensity (until about 60% of the maximal) generated almost the same size of responses (around 95–100% of the maximal response) in 14 cells tested. In the PV → Pyr connection shown in Fig. 2a, the peak amplitude, the rising slope, decay tau and total charge of the pIPSCs was 636 pA, 102 pA/ms, 42 ms and 31 pC, respectively. To demonstrate the difference from the uIPSCs, the trace of uIPSCs recorded from the same Pyr cell as shown in Fig. 1a was superimposed with that of pIPSCs on the same scale (Fig. 2a). In SOM → Pyr connections activation of a population of SOM neurons by blue laser also generated huge pIPSCs. In the same Pyr cell as shown in Fig. 1b, the peak amplitude, rising slope, decay tau and total charge of currents were 520 pA, 67 pA/ms, 101 ms and 55 pC, respectively (Fig. 2b, swollen trace). In the group analysis, the mean peak amplitude and rising slope of pIPSCs of the 11 SOM → Pyr connections (706 ± 65 pA and 102.6 ± 9.8 pA/ms, respectively) were not significantly different from those of the 12 PV → Pyr connections (632 ± 28 pA and 97.4 ± 7.3 pA/ms, respectively) (Fig. 2c,d). The mean decay tau of the pIPSCs of SOM → Pyr connections (68.0 ± 7.4 ms) was significantly (*p* < 0.01, unpaired *t*-test) longer than that of the PV → Pyr connections (35.9 ± 2.7 ms) (Fig. 2e). Also the total charge of currents of the SOM → Pyr connections (53.1 ± 8.3 pC) was significantly (*p* < 0.05, unpaired *t*-test) larger than that of the PV → Pyr connections (30.9 ± 2.3 pC) (Fig. 2f). To confirm that photo-induced pIPSCs were dependent on action potentials of presynaptic interneurons, we applied tetrodotoxin (TTX) after recording control pIPSCs in slice preparations. As will be described later, photo-stimulation generated pIPSCs in slice preparations in the same way as in the *in vivo* preparations. We observed that TTX completely blocked pIPSCs, further confirming the action potential-dependency of pIPSCs (Supplementary Fig. S3).

**Figure 3:**
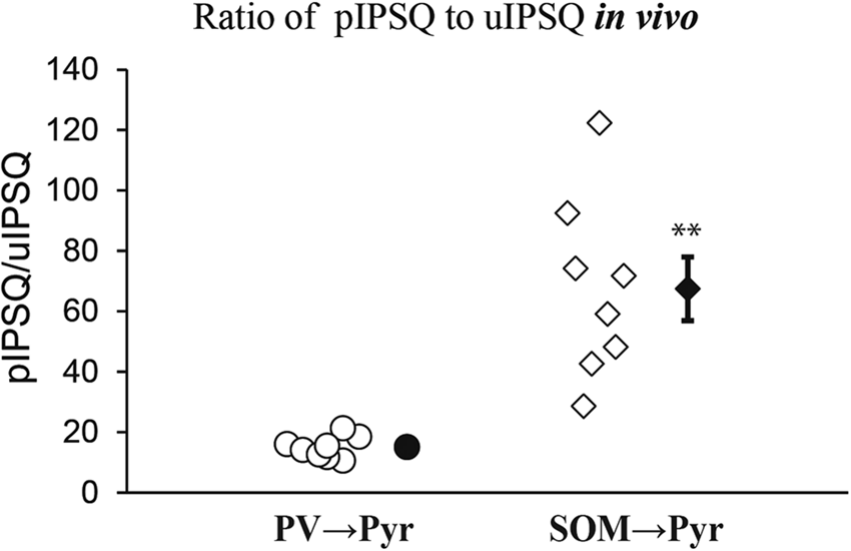
Ratio of the total charge of pIPSC (pIPSQ) to that of uIPSC (uIPSQ). Each symbol represents the ratio value for a pair of PV → Pyr cells (left column) and of SOM → Pyr cells (right column) obtained from the *in vivo* cortex. Filled symbol with vertical bar in the right represents means ± SEM. In the left column the value of SEM was very small so that it did not appear outside the symbol. Double asterisks indicate that the difference in the mean between the left and right columns is statistically significant at *p* < 0.01 (unpaired *t*-test).

Because the total charge of pIPSC (pIPSQ) is assumed to be an integral of uIPSQ of each connection in case of linear summation, we calculated the ratio, pIPSQ/uIPSQ, for each pyramidal cell to approximately assess the number of interneurons of the same subtype that functionally connects with a postsynaptic Pyr cell. The values of the 8 PV → Pyr connections were densely distributed near the mean of 14.2 ± 1.8, while those of the 8 SOM → Pyr connections were distributed in a scattered manner from 29 to 122 with the mean of 63.8 ± 11.4 (Fig. 3). In this bulk stimulation the large number of interneurons were simultaneously activated, and consequently the late accumulation of Cl^−^ was assumed to take place in postsynaptic Pyr cells. This might reduce the charge currents of pIPSCs, in particular in the late phase, as reported previously^25^ and thus lead to an underestimate of the number of interneurons. Also there is another possibility that the same group of interneurons that have mutual connections (a kind of disinhibitory connection)^26–29^ might also be activated, and consequently the number of interneurons to a postsynaptic Pyr cell may be underestimated in the present study. These points will be discussed later.

In the present study we did not analyze the other subtypes of interneurons such as serotonin receptor 3a (5HT3aR)-expressing or Vasoactive Intestinal Polypeptide (VIP)-expressing neurons^7,8,11–16,30^. To assess the contribution of the subtypes of interneurons other than PV and SOM neurons to all inhibitory activities, we measured pIPSCs of Pyr cells evoked by photoactivation of whole GABAergic neurons in VGAT-ChR2-YFP mice and compared these values with those of PV neuron- and SOM neuron-induced pIPSCs (Supplementary Fig. S4). The mean total charge of pIPSCs evoked by the activation of all GABAergic neurons was 87.4 ± 5.0 pC. The sum of the mean values of the PV- and SOM neuron-induced pIPSCs (53.1 + 30.9 = 84.0 pC) was 96% of that value. These results suggest that the contribution of the subtypes of interneurons other than PV and SOM neurons to inhibition of Pyr cells may be minor, although we cannot exclude the possibility of underestimate because of involvement of disinhibitory connections^26–29,31–34^ or shunting effects of inhibitory synapses^35,36^.

### Activation of interneurons in a large area of the cortex in slice preparations

**Figure 4:**
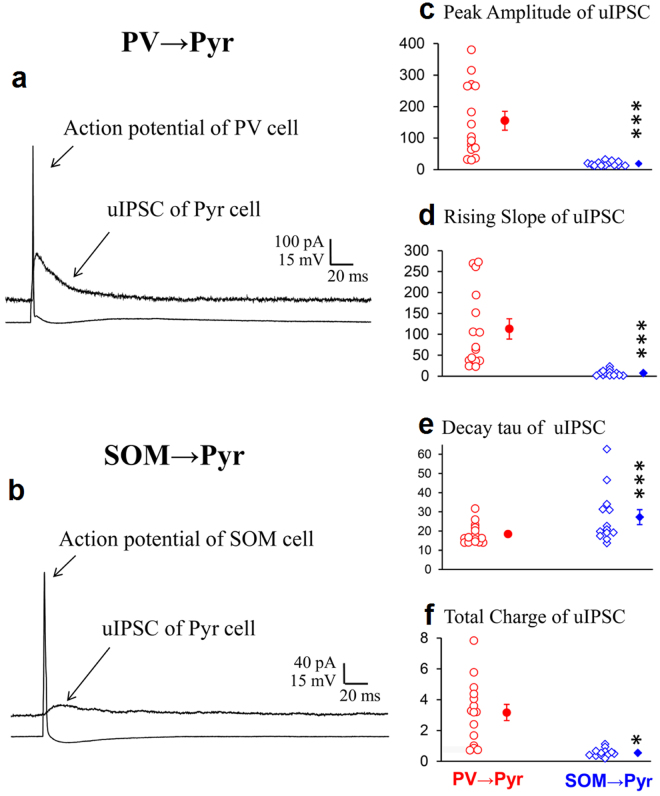
uIPSCs of Pyr cells evoked by action potentials of PV or SOM neurons *in vitro*. (**a**,**b)** uIPSCs superimposed with action potential of a presynaptic PV and SOM neurons, respectively, recorded from Pyr cells in the slice preparation. Other conventions are the same as in Fig. 1a and b. (**c**–**f**) Peak amplitude, rising slope, decay tau and total charge of uIPSCs evoked by action potentials of PV neurons (left) and those evoked by action potentials of SOM neurons (right). Triple and single asterisks indicate that the difference in the mean between the left and right columns is statistically significant at *p* < 0.001 and *p* < 0.05, respectively (unpaired *t*-test).

In the *in vivo* condition blue laser emitted through the two-photon microscope system was localized to the volume of about 200 × 200 × 400 μm of the cortex so that possible connections from interneurons outside this area might elude activation. To overcome this possible problem we activated PV or SOM interneurons with diffuse blue light which covered the volume of about 5,500 × 5,500 × 300 μm of the sliced cortex. Initially we recorded uIPSCs of Pyr cells evoked by single action potentials of PV neurons or SOM neurons in the same way as in the *in vivo* experiments. As demonstrated in the *in vivo* cortex, uIPSCs of PV → Pyr cell connections were relatively large while those of SOM → Pyr cell connections were small (Fig. 4a,b). The peak amplitude, the rising slope, the decay tau and the total charge of currents of uIPSC shown in Fig. 4a were 182 pA, 106 pA/ms, 23.1 ms and 3.2 pC, respectively. The respective values of uIPSC shown in Fig. 4b were 19.9 pA, 2.6 pA/ms, 46.6 ms and 1.1 pC. The differences in uIPSCs between the two types of connections were confirmed again by the group analysis (Fig. 4c–f). The mean values of the peak amplitudes, rising slope, decay tau and total charge of currents were 155.1 ± 7.7 pA, 112.9 ± 6.2 pA/ms, 18.4 ± 1.4 ms and 3.17 ± 0.14 pC, respectively for the 15 PV → Pyr cell connections and 18.8 ± 1.9 pA, 7.3 ± 1.9 pA/ms, 27.3 ± 3.9 ms and 0.55 ± 0.07 pC, respectively for the 13 SOM → Pyr cell connections.

**Figure 5:**
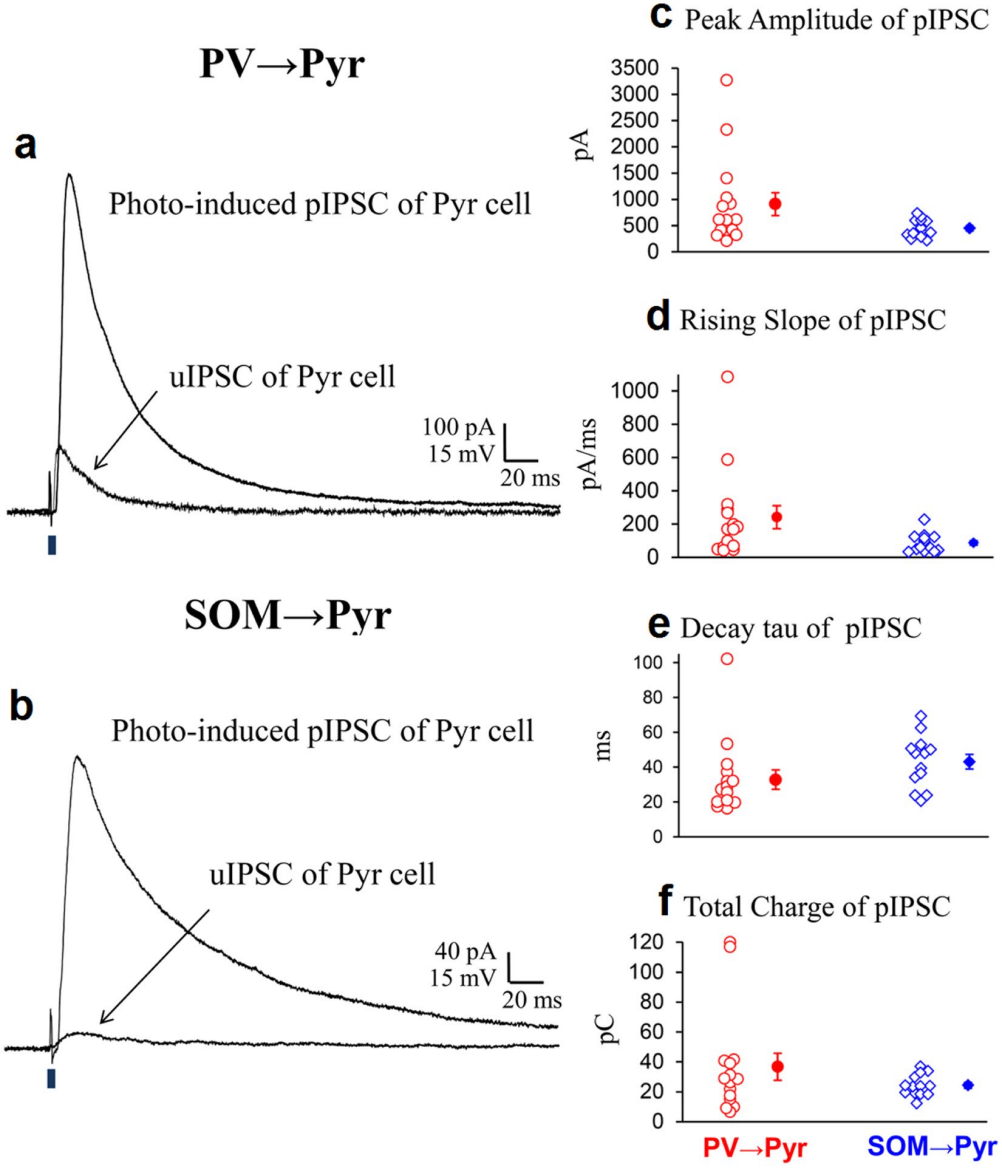
pIPSCs evoked by photoactivation of a mass of PV or SOM neurons in slice preparations. (**a**,**b**) pIPSCs recorded from the same Pyr cells as in Fig. 4a and b, respectively, so that the traces are superimposed with the uIPSCs shown in Fig. 4, respectively. (**c**–**f**) Peak amplitude, rising slope, decay tau and total charge of pIPSCs of 15 Pyr cells evoked by the mass activation of PV neurons (left) and those of another 13 Pyr cells evoked by the mass activation of SOM neurons (right).

**Figure 6:**
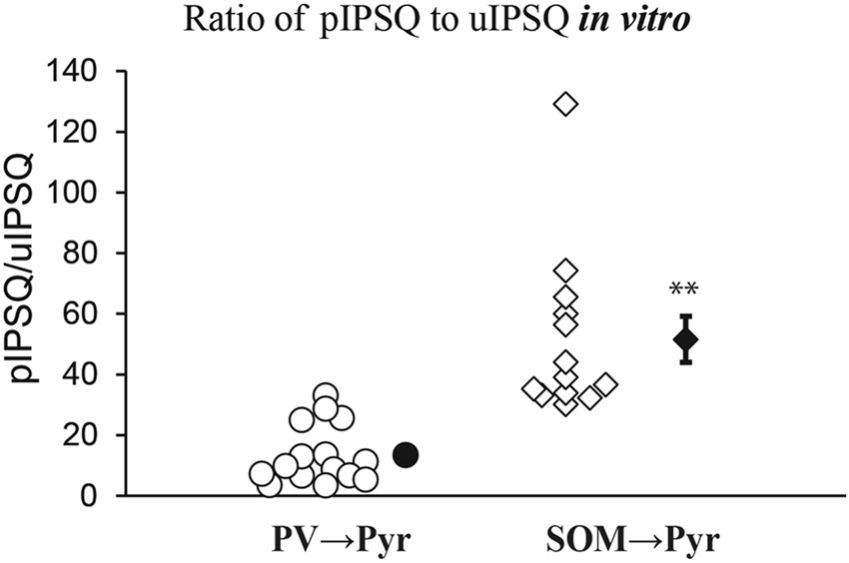
Ratio of the total charge of pIPSC (pIPSQ) to that of uIPSC (uIPSQ) in slice preprations. Each symbol represents the ratio value for the PV → Pyr cell connection (left column) and of SOM → Pyr cell connection (right column) obtained in the *in vitro* conditions. Filled symbol with vertical bar in the right represents means ± SEM. In the left column the value of SEM was very small so that it did not appear outside the symbol. Double asterisks indicate that the difference in the mean between the left and right columns is statistically significant at *p* < 0.01 (unpaired *t*-test).

Then we applied diffuse light to the sliced cortex. It induced large pIPSCs of the similar magnitude in both connections to those induced by blue laser in the *in vivo* cortex (Fig. 5a,b, swollen traces). The peak amplitude, the rising slope, decay tau and total charge of the pIPSCs of the PV → Pyr cell connection was 916 pA, 169 pA/ms, 42 ms and 41 pC, respectively (Fig. 5a). Those of the SOM → Pyr cell connection was 392 pA, 43 pA/ms, 69 ms and 34 pC, respectively (Fig. 5b). The group analysis also showed the results similar to those of the *in vivo* experiments. The mean peak amplitude, rising slope, decay tau and total charge of pIPSCs were 913 ± 57 pA, 241 ± 18 pA/ms, 32.8 ± 5.6 ms and 36.7 ± 2.3 pC for the 15 PV → Pyr connections, respectively and 447 ± 47 pA, 86.6 ± 15.7 pA/ms, 43.1 ± 4.2 ms and 24.3 ± 2.0 pC, respectively for the 13 SOM → Pyr connections (Fig. 5c–f). Then we assessed the number of PV and SOM neurons that functionally connected with a postsynaptic pyramidal cell by calculating the ratio of pIPSQ to uIPSQ for each pyramidal cell in the same way as in the *in vivo* experiments. The mean ratio of the 15 PV → Pyr connections was 13.5 ± 0.7 while that of the 13 SOM → Pyr connections was 51.6 ± 7.6 (Fig. 6). These values were very close to those obtained in the *in vivo* preparations (Fig. 3).

### Activation/suppression of single PV neurons modifies visual responses of Pyr cells

**Figure 7:**
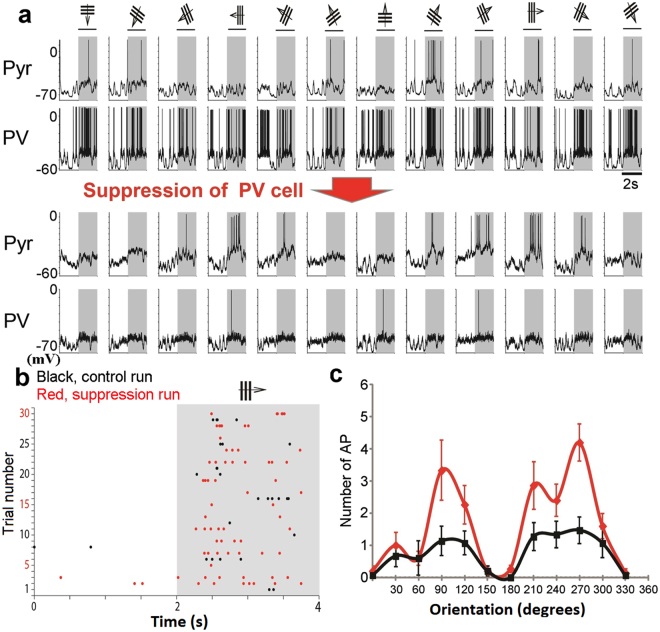
Suppression of single PV neurons enhances visual responses of postsynaptic Pyr cells. (**a**) Membrane potentials of a Pyr cell (top row) and a presynaptic PV neuron (second row) to moving grating stimuli as shown at top. Stimuli were given in the period indicated by hatched area. Membrane potentials of the Pyr cell and the PV neuron during suppression of the latter neuron are shown in the third and fourth rows. (**b**) Raster plot of action potentials of the Pyr cell before (black dots) and during (red dots) suppression of the PV neuron. (**c**) Orientation tuning curves of the visual responses of the Pyr cell before (black) and during (red) the suppression of the PV neuron. Vertical bars indicate mean ± SEM of number of action potentials (APs) in 15 responses at each orientation.

Next we tested whether activation of single interneurons modifies visual responses of postsynaptic Pyr cells. We found that suppression of a single presynaptic PV neuron enhanced visual responses of a postsynaptic Pyr cell (Fig. 7). In this pair a postsynaptic Pyr cell showed weak responses to left-tilted and vertical grating stimuli (Fig. 7a, top row) while a presynaptic PV neuron showed non-selective responses to all orientations of stimuli (second row). Then the presynaptic PV neuron was suppressed by injecting hyperpolarizing currents so that no detectable visual responses were induced (bottom row). In response to this suppression of the presynaptic PV neuron, visual responses of the postsynaptic Pyr cell were enhanced (third row). This was confirmed in the raster plot of spikes of the Pyr cell (Fig. 7b) and the tuning curve of visual responses (Fig. 7c).

**Figure 8:**
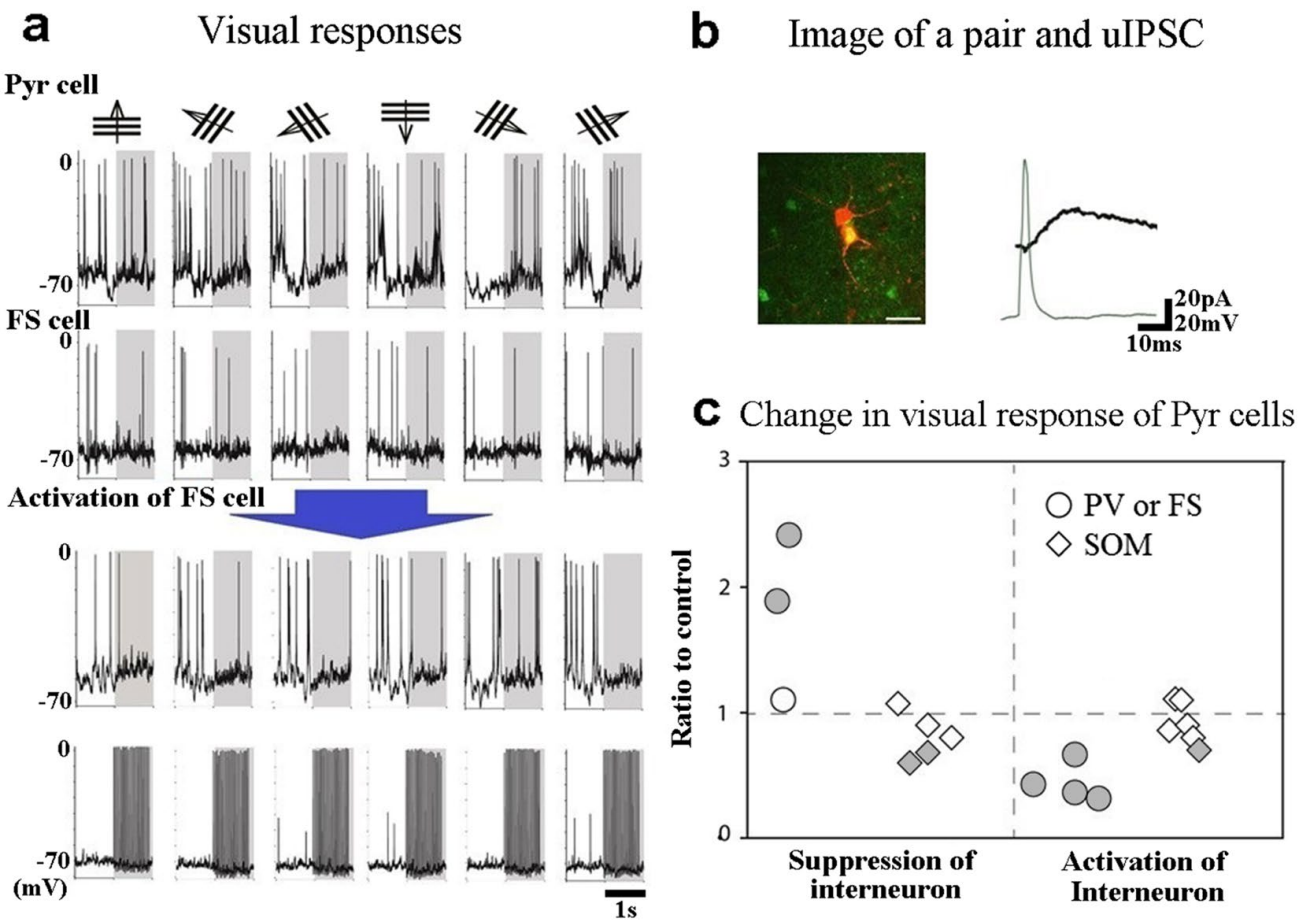
Single PV neurons modify visual responses of postsynaptic Pyr cells while single SOM neurons mostly do not. (**a)** Membrane potentials of a Pyr cell (top row) and a presynaptic interneuron of the FS type (second row) to moving grating stimuli as shown at top. Stimuli were given in the period indicated by hatched area. Membrane potentials of the Pyr cell and the FS cell during activation of the FS cell are shown in the third and fourth rows. In the fourth row injection of depolarizing current induced repetitive generation of action potentials which are truncated. Scales at the right end of the bottom row apply all rows. (**b**) Single action potentials of the FS cell and evoked uIPSCs of the Pyr cell are superimposed. Scales of 20 pA and 20 mV apply to synaptic currents of the Pyr cell and membrane potentials of the FS cell, respectively. Arrangement of the two cells is shown in inset. Scale at bottom, 20 μm. (**c**) Changes in visual responses of postsynaptic Pyr cells by suppression (shown in the left side) and activation (right side) of presynaptic interneurons of the indicated types. Ratios to the control values were calculated by the number of action potentials or the mean value of depolarization during visual stimulation. The statistical analysis with *t*-test or Mann–Whitney U test was made for each pair by comparing the values obtained during suppression or activation of presynaptic interneurons with those before the presynaptic manipulation. Cells that showed the statistically significant change are indicated by shaded symbols. The mean for the PV/FS → Pyr cell pairs was 1.8 ± 0.4 in the suppression experiment and 0.4 ± 0.01 for the activation experiment. The mean for the SOM → Pyr cell pairs was 0.8 ± 0.1 in the suppression experiment and 0.9 ± 0.1 for the activation experiment. These data were obtained from 4 PV-ChR2-YFP, 3 VGAT-ChR2-YFP and 11 SOM-ChR2-YFP mice.

In the present study we included the fast-spiking type of GABAergic neurons (FS cells) recorded from VGAT-ChR2-YFP mice in the PV neuron group, because FS cells in cortical layer 2/3 were reported to be PV neurons^8,11,12,16,37^. Then we found that activation of a single presynaptic FS cell suppressed visual responses of a postsynaptic Pyr cell (Fig. 8a). The monosynaptic connection of these cells was confirmed by generation of short-latency uIPSC of the Pyr cell following an action potential of the FS cell (Fig. 8b). In the present *in viv*o experiments it was extremely difficult to record a complete set of visual responses from synaptically connected pairs of presynaptic interneurons and postsynaptic Pyr cells. In 17 of the 35 mice altogether, we could not find functionally connected pairs. In the other 18 mice we successfully obtained a complete set of records from 3 connected pairs of PV/FS and Pyr cells and 5 connected pairs of SOM and Pyr cells in the experiments in which presynaptic interneurons were suppressed and from 4 connected pairs of PV/FS and Pyr cells and 6 connected pairs of SOM and Pyr cells in the experiments in which presynaptic interneurons were activated. In each pair we made the statistical analysis by comparing the mean number of spikes or the mean value of visually evoked depolarization during the suppression/activation of presynaptic interneurons with those before the presynaptic manipulation. In the suppression experiments the significant enhancement of visual responses was observed in two of the three PV/FS and Pyr cell pairs whereas the significant change was not observed in three of the five SOM and Pyr cell pairs (Fig. 8c, left half). In the activation experiments, the significant change was obtained in all of the four PV/FS and Pyr cell pairs whereas in only one of the six SOM and Pyr cell pairs (Fig. 8c, right half). In sum, the suppression or activation of single PV/FS neurons affected activities of almost all of the postsynaptic Pyr cells whereas that of single SOM neurons was not effective in most cases.

### No significant effect of changes in membrane potentials of interneurons on neighboring interneurons

Since PV or FS cells are reported to connect with each other through gap junctions^37–41^, activation or suppression of a single interneuron by current injections might affect neighboring interneurons so that the observed effects might not be simply ascribable to manipulation of single cell activities. To test this possibility we recorded membrane potentials of a pair of PV cells which were located closely. Currents were injected into one of the pair to hyperpolarize membrane potentials. No detectable change was observed in the neighboring PV cells during injection of currents (Supplementary Fig. S5). The same results were obtained in three pairs tested in the present study.

## Discussion

It is well known that axons of PV neurons mostly make synaptic contacts with the soma or proximal dendrites of Pyr cells while those of SOM neurons mainly target distal dendrites^8,16,23,40,42–46^. Thus it is possible that the difference in the properties of uIPSCs between PV → Pyr and SOM → Pyr cell connections might be simply ascribable to the regional difference of GABAergic synapses: synaptic currents generated at distal dendrites might elicit small IPSCs at the soma because of cable decay of currents. In this context we would like to point out that we could measure very small uIPSCs by averaging many traces (usually 15–30 traces). For example, we reliably measured uIPSCs as small as 10 pA amplitude from SOM-Pyr cell connections. This value was still larger than the noise level of our electrophysiological system for voltage-clamp recording (around 2–3 pA). Thus it is unlikely that we missed very small IPSCs. Nevertheless we cannot completely exclude the possibility that inhibitory synaptic connections which were located far from the soma or had the low release probability of GABA might be overlooked. In excitatory connections in the hippocampus it was reported that proximal and distal glutamatergic synaptic events had the similar impact on neuronal outputs, independent of their spatial location^47^, although in the neocortex more distal excitatory inputs to pyramidal cells generated the lower amplitude of somatic EPSPs^48^. The distance-dependent scaling of synaptic currents along dendrites of hippocampal pyramidal neurons was shown to have a structural basis such as synapse number and AMPA receptor expression in their dendrites^49^. In some GABAergic connections, however, the distance-dependent scaling was not seen in inhibitory synaptic currents in hippocampal pyramidal cells^50,51^. In the neocortex there has been no report on the presence or absence of the distance-dependent scaling of IPSCs to the best of our knowledge. Thus we cannot exclude the possibility of the regional difference of GABAergic synapses as a reason for the difference in uIPSCs between the two types of connections. It is also possible, however, that SOM neurons effectively affect somatic action potential generation of Pyr cells in case a certain number of SOM neurons become synchronously active. In fact, we observed that the mean peak amplitude and rising slope of pIPSCs evoked by photoactivation of a mass of SOM neurons were not significantly different from those by the mass activation of PV neurons. If the distance from the soma solely determines the magnitude of IPSCs, the former currents should have been smaller and slower-rising. However, this was not the case. Thus, one should consider the number of active connections in addition to the cable decay of IPSCs to fully understand the function of PV → Pyr and SOM → Pyr cell connections.

As mentioned in the Results section there is a possibility that activation or suppression of a single cell by current injections might affect neighboring cells so that the observed effects might not simply be ascribable to manipulation of single cell activities. However, we did not observe a detectable change in the neighboring interneurons during injection of hyperpolarizing or depolarizing currents. In our previous study only 3 of the 30 FS-FS cell pairs in layer 2/3 of the mouse visual cortex showed weak electrical coupling^52^. In the somatosensory cortex of adult mice it was reported that 14 of the 23 pairs were electrically coupled, but their coupling coefficient was only 1.43%^41^. Therefore, the possibility that our activation or suppression of single presynaptic PV cells with current injection affected activities of neighboring PV cells seems low, although cannot completely be excluded in the present study. Also there is a possibility that the manipulation of a single SOM neuron affected activities of neighboring PV cells directly through gap junctions. This possibility seems low, because electrical coupling in the neocortex was detected almost exclusively between GABAergic neurons belonging to the same class^37–41^.

In spite of the notable differences in the four parameters of uIPSCs between PV → Pyr and SOM → Pyr cell connections, optically induced pIPSCs were not significantly different from each other in the slice preparations and slightly different only in their decay tau and total charge of currents in the *in vivo* preparations. In the slice preparations the recording was carried out at the low temperature (room temperature, 23–25 °C). Thus in the physiological condition the decay of SOM-induced pIPSCs might be longer than that of PV-induced pIPSCs, which makes the charge of currents of the former pIPSCs larger. This might reflect filtering property of distantly located synapses. This possibility is unlikely, however, because the peak amplitude and rising slope was not different from those of PV-induced currents. The slow decay of SOM-induced pIPSCs might be ascribable to the convergence of large number of connections.

In the *in vivo* condition the activation of ChR2-expressed interneurons by blue laser was limited to the volume of about 200 × 200 × 400 μm of the cortex. In the *in vitro* condition, on the other hand, the activation by diffuse blue light covered the volume of about 5,500 × 5,500 × 300 μm of the sliced cortex. Nevertheless it is possible that connections from interneurons outside this area might be missed from activation. In particular, some long range connections were sectioned in slice preparations. It was reported, however, that every potential presynaptic inhibitory input to Pyr cells was detected in slice preparations of the mouse frontal and somatosensory cortices^23^. In the present *in vivo* experiments it is possible that PV or SOM neurons whose soma were located outside of the irradiated area were also activated by blue laser because their dendrites also expressed ChR2 (see inset pictures of Fig. 1 and Supplementary Fig. S2). Previous reports demonstrated that dendrites of PV and SOM cells in cortical layer 2/3 were distributed more than a few hundred μm from their soma^45,53,54^. It is also to be taken into consideration that ChR2 in interneurons is expressed also in axons, and axon terminals of cortical pyramidal cells can be activated by blue laser in the mouse neocortex^55^. In hippocampal slices it was reported that axonal activation required much brighter or stronger light than soma activation: The spike generation probability was already saturated at the soma whereas still near zero at axons at the laser intensity of 18 mW/mm^2^, the same intensity as ours^56,57^. These results suggest that the possibility of activation of axons in the present experiments may be low. Also it is to be considered that postsynaptic Pyr cells extend their basal dendrites about 150 μm from the soma in the horizontal direction^58^. It is possible, therefore, that our blue laser activated interneurons whose soma were located far from postsynaptic Pyr cell soma, but still induced IPSCs in the Pyr cells. Consequently the extent of activated area by blue laser in the *in vivo* condition may be similar to that by diffuse blue light in slice preparations. We believe that this is a reason why the numbers of PV and SOM cells that were assessed to be functionally connected with postsynaptic Pyr cells in the *in vivo* preparations were almost the same as those in the slice preparations.

The present assessment of the number of presynaptic interneurons to postsynaptic Pyr cells is based on the assumption that the charge currents are linearly summated, as mentioned in the Results section. However, there is a possibility that the activation of many interneurons will affect the summation of the inhibitory events in Pyr cells. For example, a massive activation of multiple GABAergic synapses results in intracellular Cl^−^ accumulation which may lead to a depolarizing shift of E_GABAA_. In fact, it was reported that the direction of GABA_A_ receptor currents was changed from outward to inward after prolonged Cl^−^ influx^25^. In the present study, however, it is to be noted that interneurons were not sequentially but simultaneously activated so that the initial phase of photo-induced IPSCs was probably not affected by the delayed accumulation of Cl^−^, although the late phase of IPSCs was likely to be affected. Thus we cannot completely exclude the possibility that the total charge of inhibitory currents might be underestimated to some degree. Also there is another possibility that the shunting effects of inhibitory synapses might reduce pIPSCs. The present results that the rising slopes of the pIPSCs were much slower than those of uIPSCs (for *in vivo* and *in vitro* recordings, for PV and SOM cells) might suggest such a shunting effect in the massive activation of interneurons. Therefore, the present calculation may give an estimate of the minimum number of interneurons that converge to postsynaptic Pyr cells. Although we cannot exclude such an underestimate of the number of interneurons, the large difference in uIPSCs albeit the similar size of pIPSCs between the PV → Pyr and SOM → Pyr connections suggests that the number of each subtype of interneurons functionally connecting to postsynaptic Pyr cells is notably different.

Recently it was reported that 18% of reporter fluorescence protein-expressing interneurons in SOM-Cre mice^59^ crossed with Ai14 reporter line displayed electrophysiological fingerprint of FS interneurons^60^, suggesting that a proportion of PV neurons might be included in interneurons classified as SOM neurons in SOM-Cre mice in the present study. In the present study we analyzed the cortical tissue with immunohistochemistry, and demonstrated that 96–97% of ChR2-YFP expressing interneurons were immunoreactive for SOM, as shown in Supplementary Fig. S2. Thus the possibility of the false-positive contamination of interneurons is quite low, although cannot completely be excluded.

In sum, the present findings suggest that there is a much greater functional convergence of SOM cells than PV cells to postsynaptic Pyr cells and a small number of PV cells or even a single PV cell affects visual responses of target Pyr cells whereas a larger number of SOM cells do so when they work together in a mass.

## Methods

### Animal preparations

All experimental procedures were approved by the Animal Experiment Committee of RIKEN Brain Science Institute and the experiments were carried out in accordance with the guidelines of the Committee. The following three types of mice in which PV, SOM or whole GABAergic interneurons express ChR2, of either sex, were used at postnatal days 45–70 (P45-70). 1. PV-ChR2-YFP mice, B6;129P-Pvalbtml (cre) Arbr/J × B6;129S-*Gt(ROSA)26Sor* ^tm32(CAG-COP4*H134R/EYFP)Hze^ */J*, 2. SOM-ChR2-YFP mice, STOCK Sst < Tm2.1(cre)Zjh > J × B6;129S-*Gt(ROSA)26Sor* ^tm32(CAG-COP4*H134R/EYFP)Hze^ */J*, 3. VGAT-ChR2-YFP mice, B6.Cg-Tg(Slc32a1-COP_4_*H_134_R/EYFP)8Gfng/J. The animals were anesthetized with urethane (1.5–1.9 mg/g body weight), supplemented with additional doses of urethane when necessary. The level of anesthesia was continuously monitored by observing heart and respiration rates, which were not changed in response to a strong touch of the tail. A small custom-made head chamber to stabilize the animal’s head under a two-photon microscope was attached over the occipital region of the left hemisphere. The part of the skull and dura mater over the primary visual cortex were removed, and the exposed cortex was covered with agarose (1.5–2.0% in Ringer’s solution).

*In vitro* experiments PV-ChR2-YFP or SOM-ChR2-YFP mice of either sex were used at P25-35. For slice preparations, animals were anesthetized with Isoflurane (Abbott, Abbott Park, IL) and then decapitated. The brains were rapidly removed and placed in the cold oxygenated artificial cerebrospinal fluid (ACSF). Coronal slices of the visual cortex (300 µm thick) were obtained using a tissue slicer (Vibratome 3000). Slices were placed in an incubating chamber filled with oxygenated ACSF at 31 °C for 30 min and then stored at room temperature (23–25 °C) until transferred to a submerged recording chamber. The recording was carried out at room temperature. The ACSF had the following composition (in mM): NaCl, 124; KCl, 3.0; CaCl_2_, 2.0; MgCl_2_, 1.0; NaH_2_PO_4_, 1.25; NaHCO_3_, 26.0; and glucose, 10.0, at pH 7.4. The ACSF was bubbled continuously with 95% O_2_–5% CO_2_. The flow rate of the ACSF was about 2.5 ml/min. An antagonist for NMDA receptors, DL-2-amino-5-phosphonovaleric acid (APV, Sigma-Aldrich) at 100 µM and an antagonist for AMPA receptors, 6-cyano-7-nitroquinoxaline-2,3-dione (CNQX, Sigma-Aldrich) at 20 µM were added to the ACSF. In part of the experiments tetrodotoxin (TTX, Wako Pure Chemicals) at 1 µM was added to the ACSF.

### Visualization of neurons under the *in vivo* two-photon laser scanning microscopy

Images of cells were visualized with an upright microscope (BX61WI, Olympus) equipped with a water immersion objective lens (XLPlan N 25X/NA 1.05w, Olympus) and the laser scanning microscope system (FV1000-MPE, Olympus), which was coupled with a mode-locked Ti:sapphire laser (MaiTai HP DeepSee, Spectra-physics, Mountain View, CA). The mode-locked lasers were set at the wavelength of 800 and 930 nm for excitation of Alexa 594 which was injected into target neurons and Venus/YFP which were expressed in a given type of interneurons, respectively. Emitted fluorescence was divided into long wavelength (>570 nm) and short wavelength light with a dichroic mirror (570 nm, Olympus), and short-wavelength light was further filtered through a bandpass filter (510–550 nm, Olympus). Both wavelengths of emitted fluorescence were detected simultaneously using two photomultiplier tube detectors (PMTs). The microscope objective was shielded from possible stray light by covering the space over the animal’s head with lightproof cloth and clay.

### *In vivo* electrophysiology

Whole-cell current-clamp recordings of membrane potentials were performed from YFP-positive interneurons at a depth of 110–230 μm from the cortical surface. Whole-cell voltage-clamp or current-clamp recordings of membrane currents/potentials were simultaneously performed from nearby pyramidal cell-like neurons. The mean distances between the PV → and SOM → Pyr cell pairs were 28 ± 5.0 and 36 ± 6.0 μm, respectively. These recordings were performed with a pair of multi-clamp amplifier (Multiclamp 700B). Recording electrodes were pulled from borosilicate glass capillary with filaments (0.86 mm inner diameter, 1.5 mm outer diameter). The composition of the internal solution for voltage clamp recordings was as follows (in mM): CsMeSO_4_, 130; CsCl, 3; HEPES, 10; MgATP, 4; Na_3_GTP, 0.3; EGTA, 1 and Na-Phosphocreatine, 10. The resistance of the electrode filled with this solution was 3–6 MΏ. The composition of the internal solution for current clamp recordings was as follows (in mM): K-Gluconate, 130; CaCl_2_, 0.1; HEPES, 10; MgATP, 4, Na_3_GTP, 0.3; MgCl_2_, 2; Na-Phosphocreatine, 10 and EGTA, 1. The resistance of the electrodes filled with this solution was 6–8 MΏ. For recording IPSCs from Pyr cells the former CsMeSO_4_-based solution was used while the latter K-Gluconate-based solution was used for inducing action potentials of inhibitory interneurons and recording visual stimulation-induced action potentials of Pyr cells. For analyzing the morphology of recorded cells Alexa 594 was added to the internal solution at 0.025 mM. The osmolarity of the solution was 280–290 mOsm, and pH was adjusted to 7.2–7.4 with CsOH or KOH.

Whole-cell recordings from pyramidal cells were targeted to YFP-negative neurons with large soma of pyramidal shape and large apical dendrites which were visualized by filling the extracellular space with Alexa flour during shadow patching. They were confirmed to be pyramidal cells by identifying spines along dendrites visualized with intracellularly injected Alexa and regular spiking pattern evoked by depolarizing current injection. The mean resting membrane potential of Pyr cells was 70.2 ± 1.8 mV (n = 16). Action potentials were generated in YFP-positive interneurons by injection of depolarizing currents of ˂250 pA for 5 ms duration. Evoked IPSCs were recorded from pyramidal cells at the holding potential of 0 mV. Membrane currents were filtered at 2–5 kHz and sampled at 20 kHz using custom made LabView software, and fed into a computer with an NI-DAQ board (PCI-MIO-16E-4, National Instruments). Data analysis was performed using the MATLAB program. Series resistance was monitored before and after each recording session by applying small voltage steps (5 mV of 250 ms duration). The recording was excluded from further analysis if the series resistance was higher than 25 MΩ and/or changed >20% during recording.

### In slice electrophysiology

Dual whole-cell recordings were made from a pair of interneurons and Pyr cells in layer 2/3 of the visual cortex under infrared differential interference contrast optics. PV or SOM interneurons in cortical slices were visualized with an epifluorescence microscope (BX51WI, Olympus). The composition of the internal solution was the same as *in vivo* experiments. The membrane potentials were recorded with a multi-clamp amplifier (700B), filtered at 2–5 kHz and digitized at 10 kHz, and fed into a Pentium 4 personal computer with a digitizer computer interface (PCI-MIO-16E-4, National Instruments). The analysis was made using Igor 4.01 program. Cells were not used for analysis if the resting membrane potential was more positive than −50 mV, series resistance was >25 MΩ, or if any of these parameters changed by 20% during data acquisition. The mean resting membrane potentials of Pyr cells were 66.6 ± 1.3 mV (n = 28).

### Activation of given types of interneurons

To activate a particular type of interneurons we employed the two methods: Electrical stimulation of single cells under observation by current injection through recording pipettes and optical stimulation of a population of interneurons which expressed ChR2 through blue light. This optical stimulation was performed using 473 nm laser through the objective lens of the two-photon microscope in the *in vivo* preparations (1 ms pulses at 10 Hz for 2 s, 18 mW/mm^2^) or an LED source of blue light through the objective lens of the Olympus upright microscope in the slice preparations (single pulses of 1 ms duration given at 20 s intervals, 20 mW/mm^2^).

### Recordings of uIPSCs

Dual whole-cell recordings were performed from a pair of Pyr cell and interneuron to record uIPSCs evoked by single action potentials of PV, SOM or FS-GABA neurons in layer 2/3 of the visual cortex. The following parameters of uIPSCs were measured in traces obtained by averaging 15–30 consecutive responses: Peak amplitude (from the baseline to the peak of uIPSC); Rising slope (slope of a fitting line from 10–90% after onset of uIPSC); Total charge of currents as the integral of uIPSCs until the trace returned completely to the baseline; and Decay τ which was measured by fitting a single exponential to the decay phase of the uIPSCs.

### Visual stimulation

Visual stimuli were presented on an LCD monitor (Flexscan L788, 19 inch, Eizo Nanao) 30 cm from the eye of the animal, covering 80 × 50 degrees of the visual field. Square-wave gratings (0.05 cycle/degree; 10–20 degree/s) at 100% contrast were moved on the monitor in 12 directions at 30 degree steps (from 0 to 330 degrees). The visual stimulation procedure was as follows: 2 s before, 2 s during and 2 s after the presentation of visual stimulus. In the experiments in which presynaptic single cells were activated or suppressed these 12 patterns of 2 s visual stimuli were presented 4–6 times in a randomly shuffled order before, during and after activation or suppression of the presynaptic interneurons.

### Evaluation of effects of presynaptic cell activation/suppression on visual responses of postsynaptic cells

The number of action potentials during visual stimulation was counted before, during and after activation or suppression of presynaptic interneurons. If postsynaptic neurons did not generate action potentials in response to visual stimulation, the membrane depolarization during the presentation of visual stimuli moving in the same direction was averaged over trials (5–12 trials per each experiment). These visually evoked depolarizations were computed as areas under the trace of depolarization above the mean baseline level of membrane potentials in the pre-visual stimulation period. Cells were defined as visually responsive when one of the averaged signals was larger than 2 SDs of the baseline signals during the pre-stimulation period.

### Double immunofluorescence staining

The following procedure was carried out as previously reported^29^. Briefly, after fixation with 4% (w/v) paraformaldehyde in 0.1 M phosphate buffer (PB), the brain blocks were cut into 40 µm-thick coronal sections on a freezing microtome. The brain sections were incubated overnight with a mixture of chicken anti-GFP antibody (GFP-1020; Aves Labs, Tigard, OR; 20 µg/ml) and rabbit anti-PV (PV25; Swant, Bellinzona, Switzerland; 1/4000) or rabbit anti-SOM (T-4103; Peninsula Laboratories, Belmont, CA; 1/1000). The sections were further incubated overnight with a mixture of AlexaFluor488-conjugated goat antibody against chicken IgY (A-11039; Life Technologies; 5 µg/mL) and Cy3-conjugated donkey antibody against rabbit IgG (711-165-152; Jackson ImmunoResearch Laboratories; 10 µg/mL), and counterstained by NeuroTrace 435/455 blue fluorescent Nissl stain (N-21479; Life Technologies; 1/200) to identify the cytoarchitecture of the primary visual cortex.

The sections were observed under a Leica TCS SP8 confocal laser-scanning microscope (Leica) equipped with a 25 × water-immersion objective lens (HB FLUOTAR L25x/0.95 W VISIR, NA = 0.95) and HyD detectors. We acquired images at 5.0 Airy disk unit and 2048 × 2048 pixels. NeuroTrace 435/455 blue, AlexaFluor488 and Cy3 were sequentially excited with 405, 488 and 552 nm laser beam, and observed through 410–470, 495–550 and 560–610 nm emission prism windows, respectively. After taking images, we counted the number of neurons across cortical layers.

### Statistical analysis

In the present study, values are given as the mean ± SEM, unless otherwise mentioned. For statistical analysis, values between two different groups of cells were compared with unpaired *t*-test. The *t*-test was used in case the values plotted in representative neurons showed the normal distribution. Statistical evaluation of the normal distribution was made using the Kolmogorov–Smirnov test. In case the distribution of values was not normal, the Mann–Whitney U test was used.

### Data availability

The datasets generated during and/or analyzed during the current study are available from the corresponding author on reasonable request.

## Electronic supplementary material

Supplementary Information
