# Supplementary material for: Parvalbumin-expressing interneurons can act solo while somatostatin-expressing interneurons act in chorus in most cases on cortical pyramidal cells
Source: Sci Rep. 2017 Oct 6;7:12764. doi: 10.1038/s41598-017-12958-4 (PMC5630625; doi:10.1038/s41598-017-12958-4)
Supplement: Supplementary file 1 — Supplementary Information [file 41598_2017_12958_MOESM1_ESM.pdf]

Supplementary Information for

Parvalbumin-expressing interneurons can act solo while somatostatin-expressing interneurons act in chorus in most cases on cortical pyramidal cells

Mir-Shahram Safari, Javad Mirnajafi-Zadeh, Hiroyuki Hioki, Tadaharu Tsumoto*

*Corresponding author: E-mail, tsumoto@brain.riken.jp

This file includes

Supplementary Figures S1 to S5

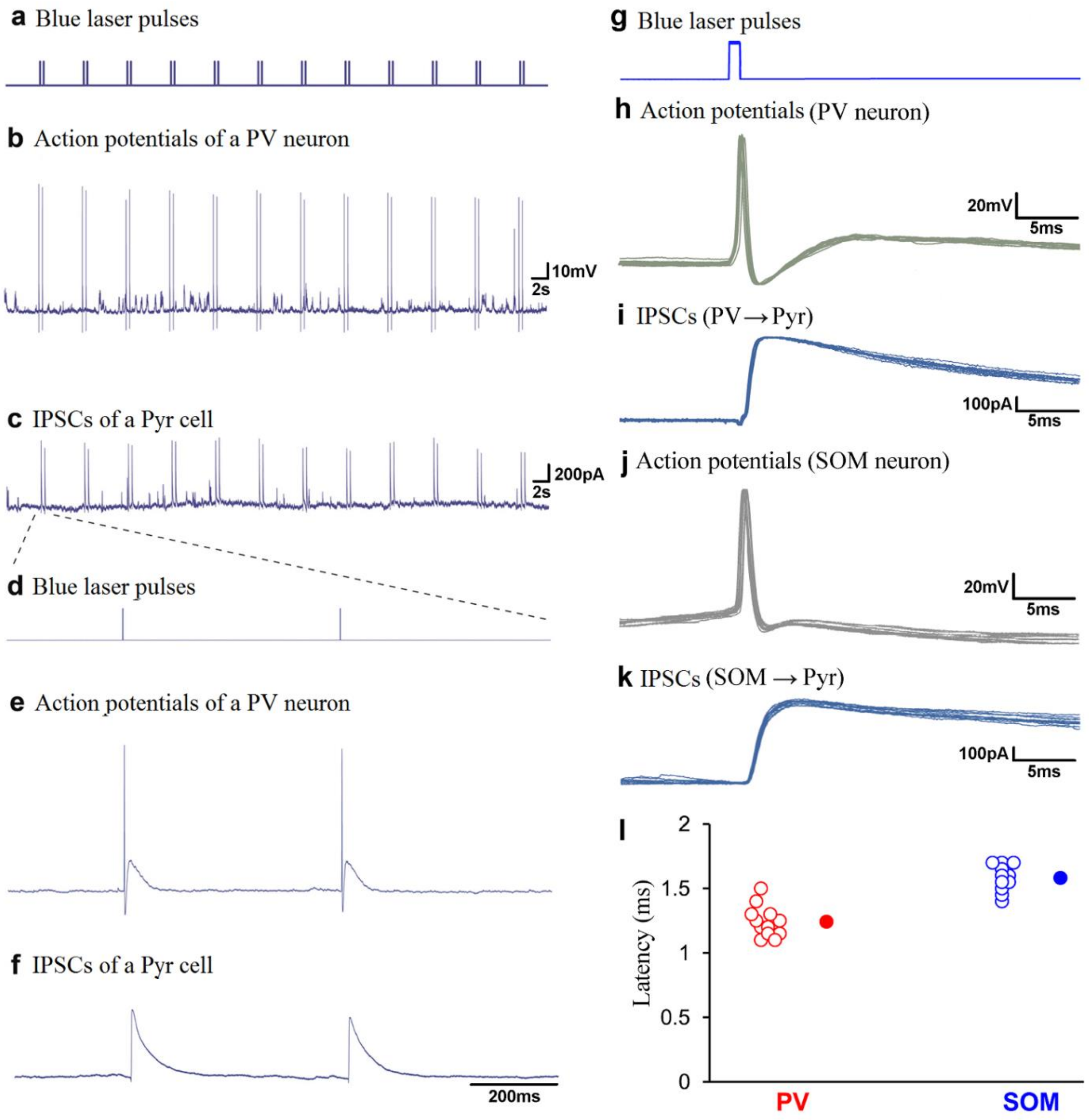

Supplementary Figure S1.

Photo-induced action potentials of interneurons and subsequently evoked IPSCs of Pyr cells. (**a**) A pair of blue laser pulses (1 ms width each at the interval of 500 ms) applied to the visual cortex at the frequency of 0.16 Hz. (**b**) Action potentials of a PV neuron which were induced by laser pulses. The intensity of laser was adjusted so that each laser pulse generated single action potentials. (**c**) IPSCs of a Pyr cell evoked by action potentials of the PV neuron. (**d-f**) Parts of the traces **a-c** are shown at the expanded time scale, as indicated by dotted lines. (**g-i**) Parts of the traces **d**, **e** and **f** are further expanded at the faster time scale. (**j**) Action potentials of a SOM neuron which were induced by blue laser. (**k**) IPSCs of a Pyr cell evoked by action potentials of the SOM neuron. Superimposition of 12 sweeps in **h-k**. (**l**) Latencies of 12 PV and 11 SOM neurons from the onset of laser pulse to the peak of action potentials. The means ± SEM are 1.24 ± 0.03 and 1.58 ± 0.03 ms for PV and SOM neurons, respectively.

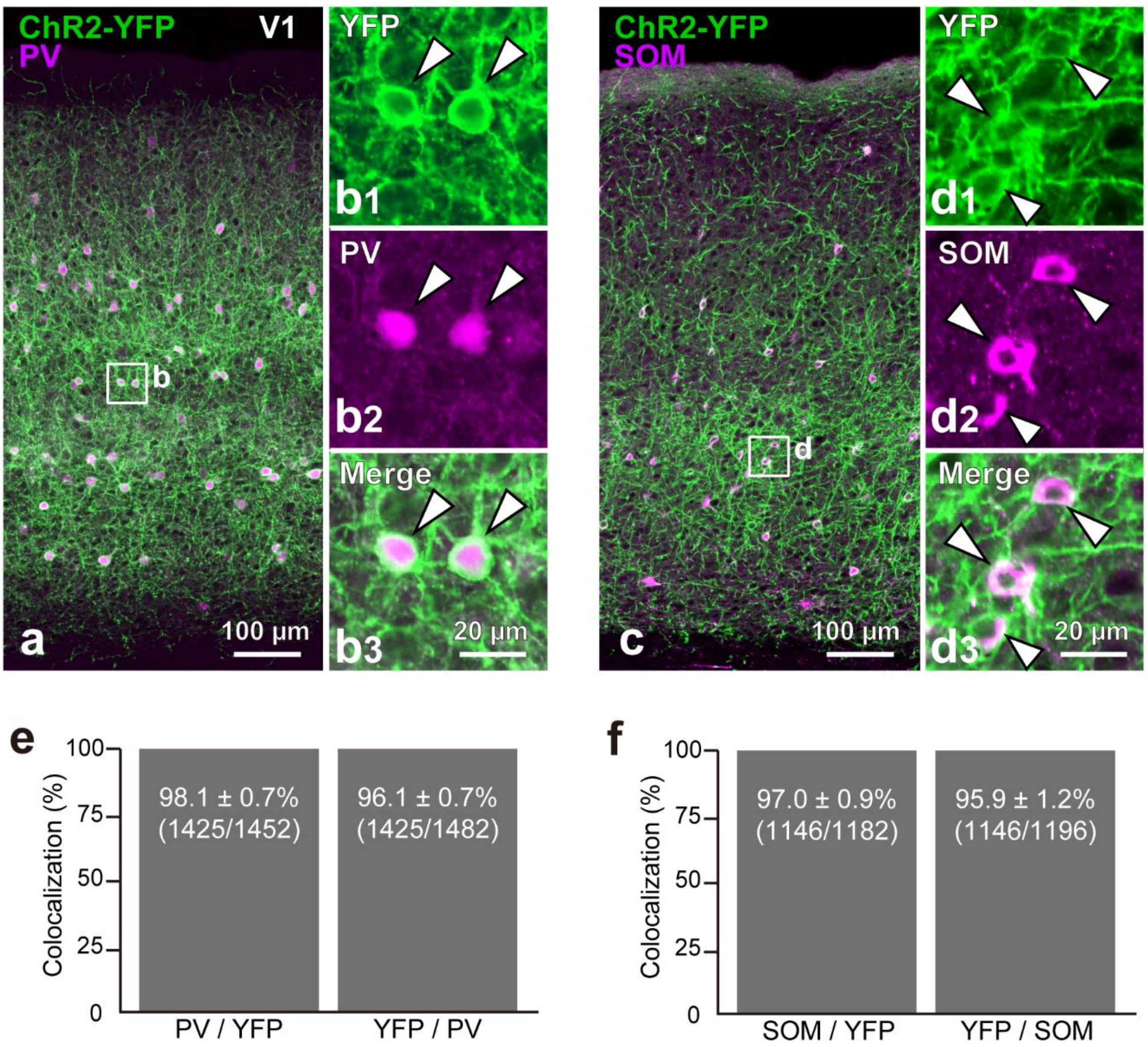

Supplementary Figure S2.

Specific expression of ChR2 in PV or SOM neurons. (**a**) The immunoreactivity for YFP and PV was visualized with AlexaFluor488 (green) and Cy3 (magenta), respectively. (**b1-3**) Magnified image of the area indicated by rectangle in **a**. (**b1**, **b2**) Stained by antibody as indicated. (**b3**) Superimposed image of **b1** and **b2**. Arrowheads indicate the co-localization. (**c**) The immunoreactivity for YFP and SOM was visualized with AlexaFluor488 (green) and Cy3 (magenta), respectively. (**d1-3**) Magnified image of the area indicated by rectangle in **c**. Other conventions are the same as in **b1-3**. (**e**) Expression of ChR2 in PV neurons. The values indicated were obtained from 9 sections of three mice. (**f**) Expression of ChR2 in SOM neurons. Other conventions are the same as in **e**.

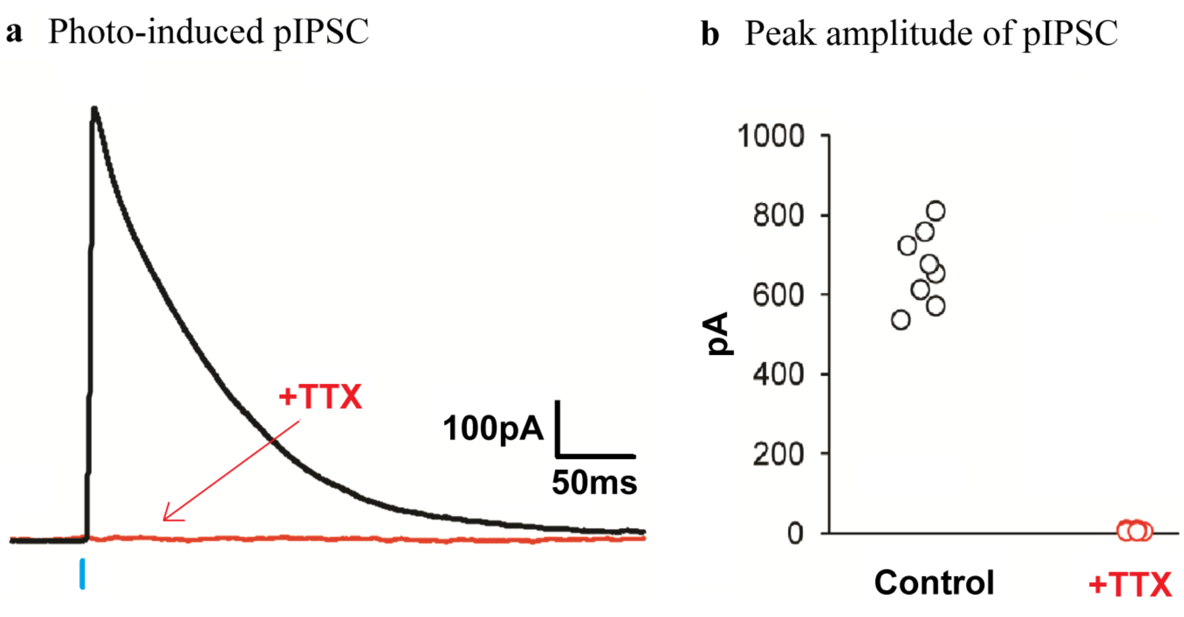

Supplementary Figure S3

Photo-induced IPSC is dependent on action potential. (**a**) Laser pulse stimulation at the 1 ms width (shown by vertical blue line at the bottom) induced IPSC of a Pyr cell (black trace) and this IPSC was completely abolished by bath application of tetrodotoxin (TTX) at 1 μM, shown by red trace. (**b**) Peak amplitude of IPSCs of 8 Pyr cells before (control) and after the application of TTX (+TTX). The means ± SEM are 667 ± 33 and 5.75 ± 0.7 pA for Control and +TTX conditions, respectively.

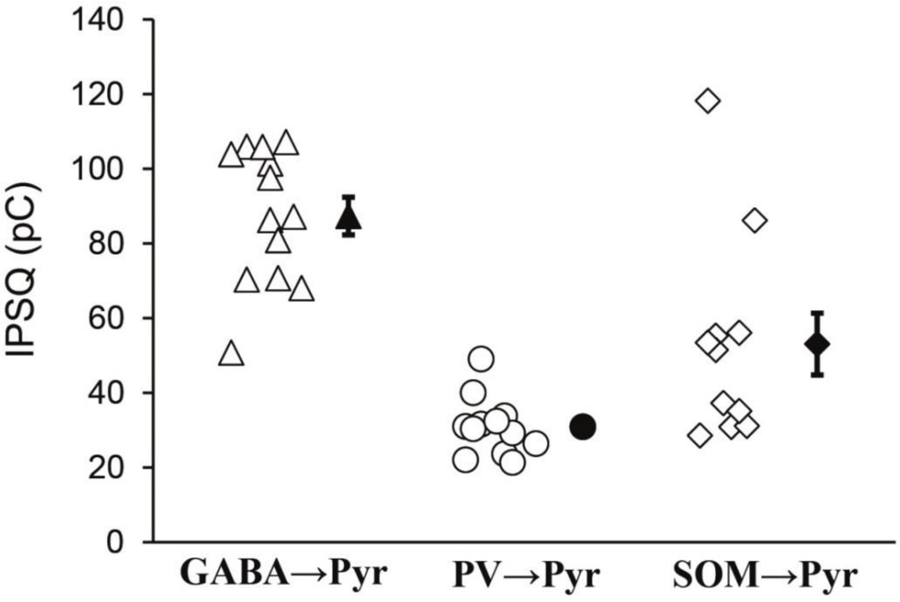

Supplementary Figure S4.

Total charge of population IPSCs of pyramidal cells. The mean total charges of currents (IPSQs) were 87.5 ± 5.0 pC (n=13), 30.9 ± 2.3 pC (n=12) and 53.1 ± 8.3 pC (n=11) for whole GABA→Pyr, PV→Pyr and SOM→Pyr connections, respectively. The data for the PV→Pyr and SOM→Pyr connections are the same as those shown in Figure 2f. The sum of the mean total charge of currents evoked from PV and SOM neurons (30.9+53.1=84.0 pC) was 96% of those from whole GABAergic neurons (87.5 pC).

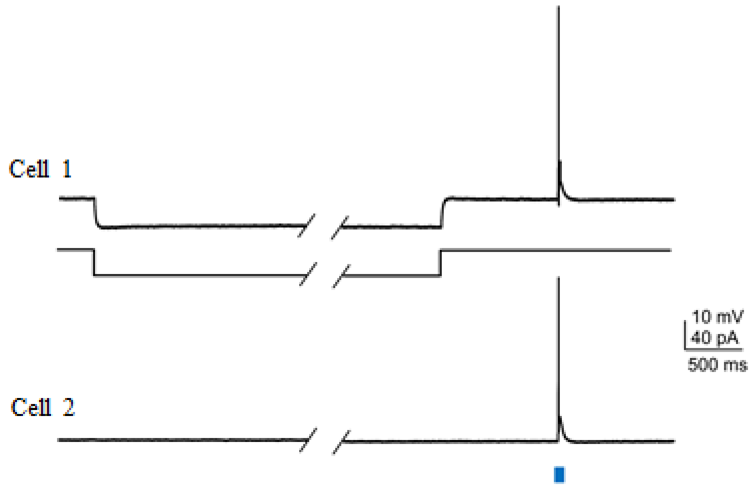

Supplementary Figure S5.

No effect of injection of hyperpolarizing currents into a PV cell on membrane potential of a neighboring PV cell. Hyperpolarizing currents of 40 pA for 20 s were injected into the cell 1. The center-to-center distance between the two cells in the sliced visual cortex was 30 µm. After stopping the current injection blue light (wave length, 473 nm) was irradiated for 1 ms to the slice containing the two cells to test the condition of the cells. Generation of action potentials confirmed that the cell 2 as well as the cell 1 were intact. The scale of 40 pA applies to the injected currents (second row) and that of 10 mV applies to the membrane potentials.
